# Visible light-induced polymerization initiated by borate salts of bicationic monochromophoric benzothiazolestyrylium dyes

**DOI:** 10.1007/s00396-014-3343-4

**Published:** 2014-08-14

**Authors:** Janina Kabatc, Katarzyna Jurek

**Affiliations:** Faculty of Chemical Technology and Engineering, University of Technology and Life Sciences, Seminaryjna 3, 85-326 Bydgoszcz, Poland

**Keywords:** Benzothiazolestyrylium dyes, Photosensitizers, Synthesis, Properties, Radical polymerization

## Abstract

A series of bicationic monochromophoric hemicyanine dyes based on benzothiazolestyrylium residues were synthesized. The dyeing photoinitiating systems consisting of *N*-[3-(4-methylpyridino)propyl]-2-(*p*-substituted styryl)benzothiazolium dihalides as chromophores and *n*-butyltriphenylborate anion as electron donor were also prepared to achieve an efficient photoinitiators for free-radical polymerization in a visible-light region. The relative photoinitiating efficiencies of novel photoinitiators of acrylate monomers polymerization were evaluated.

## Introduction

The photoinitiating system generates free radicals that initiate radical chain polymerization of an unsaturated monomer. It may be a single compound, typically called a photoinitiator rather than a photoinitiator system that absorbs light and undergoes unimolecular reaction to form radicals. It may consist, as well, several different compounds that undergo a complex series of consecutive reactions to produce the initiating radicals [1]. The photoinitiation of polymerization usually does not occur upon interaction of an excited singlet or triplet state of dye with monomer molecule [2].

Therefore, in most practical applications of photoinduced radical polymerization, the sensitizers are UV-absorbing compounds that undergo unimolecular fragmentation in an excited state to form the initiating radicals. It has been proven difficult to extend this concept very far into the visible region of the spectrum. Most of the useful initiating systems that respond to visible light are based on quite different, usually bimolecular chemistry. For example, a sensitization, in order to be useful in the blue region, involves a bimolecular electron-transfer reaction between an excited chromophore and an electron-donating molecule to form, in the case of ionic reactants, for example a radical of electron donor and a radical of electron acceptor, respectively. Subsequent reactions result in radicals that are capable of initiating polymerization [3]. There are two types of sensitization of free-radical polymerization: photoreducible (very common) and photooxidizable [2, 4].

In most cases, photoreducible dyes have maximum absorption band in a visible-light region. A suitable dyeing photoinitiating system must first exhibit a high absorption in the wavelength delivered by visible-light sources (LEDs, lasers) and, secondly, efficiently generate the reactive-initiating radicals [3]. The well-known examples of dyeing photoinitiating systems are cation-anion couples composed of hemicyanine dye/borate salt. The hemicyanine dye acts as a visible-light absorber in many photoinitiating systems. Such dyeing photoinitiating systems were first described by Schuster et al. [5, 6]. Their work [6] on the photochemistry of cyanine borates led to the preparation of color-tunable, operating in the visible-light region commercial photoinitiators [7]. According to this study, the initiation step of the reaction involves an alkyl radical formation as a result of photoinduced electron transfer from borate anion to an excited singlet state of cyanine dye, followed by the rapid cleavage of the carbon-boron bond in boranyl radical.

Application of hemicyanine dye borate salt as photoinitiating system gives opportunity of changing the usefulness region of these photoinitiators by changing the chromophore structure. For example, the modification of the hemicyanine dye structure by introduction of the second, not conjugated with the dye molecule, quaternary nitrogen atom significantly enhances the rate of photoinitiation [8].

From our studies, it is known that for cyanine borate salts, in some cases, there is a direct relationship between the rate of free-radical polymerization and the rate of electron-transfer process. On the other hand, the efficiency of electron-transfer process depends on the distance between an electron acceptor and an electron donor. Since the lifetime of an excited singlet state of cyanine dye is very short (about or less than 1 ns), therefore, the formation of the tight ion pair increases the efficiency of electron-transfer process. In the simplest way, the formation of contact ion pair can be enhanced by an increase of electron donor ion concentration (co-initiator). It is noteworthy that for cyanine borate salts, the influence of an additional amount of borate anion on the rate of photoinitiated polymerization depends on a dye cation type. So, it may be achieved by an increase of an electron donor concentration in proximity to the absorbing dye, exclusively by a coupling of electron donor. In the case of cyanine-borate couple, it is possible by adding an additional organic cation capable of an ion pair formation with borate ion.

This work presents the study on the synthesis and both photophysical and photochemical properties of new, singlet-state free-radical dyeing photoinitiating systems with enhanced photoinitiation ability caused by an artifical increase of an electron donor concentration in proximity to an absorbing chromophore. This unique and easily achieved improvement was carried out by covalent bonding of quaternary 4-metylpyridinium cation to cationic hemicyanine dye.

## Experimental

### Materials

2-Methylbenzothiazole, 1,3-dibromopropane, 4-picolin, 4-(*N*,*N*-dimethylamino)benzaldehyde, 4-(*N*,*N*-diethylamino)benzaldehyde, 4-(1-pyrrolidinyl)benzaldehyde, 4-(1-piperidinyl)benzaldehyde, formylcarbazole, and solvents were obtained from Aldrich Chemical Co. (Poland). Used as co-initiator, tetramethylammonium *n*-butyltriphenylborate (*B*) was synthesized based on the method described by Damico [9]. 2-Ethyl-2-(hydroxymethyl)-1,3-propanediol triacrylate (TMPTA) and 1-methyl-2-pyrrolidinone (MP) were purchased from Aldrich and were used as monomer and solvent, respectively.

### Measurements

The ^1^H NMR spectra were recorded with the use of a Varian spectrometer Gemini 200 operating at 200 MHz. Dimethylsulfoxide (DMSO) was used as solvent and tetramethylsilane (TMS) as internal standard. The elemental analysis was made with a Vario MACRO 11.45-0000, Elementar Analyzer. Melting points (uncorrected) were determined on the Boëthius apparatus—PGH Rundfunk, Fernsehen Niederdorf KR, Stollberg/E.

(i) All final products of synthesis were identified by ^1^H NMR spectroscopy and elemental analysis. The results obtained were the evidence that the reaction products were of the desired structures. The purity of synthesized compounds was determined using a thin layer chromatography and by measuring the melting points. The purity of the dyes was as it is required for the spectroscopic studies.
(ii) Spectroscopic measurements: UV-Vis absorption spectra were obtained using a Shimadzu UV-Vis Multispec-1500 Spectrophotometer and steady-state fluorescence using a FLS920 Edinburgh Instruments Spectrofluorimeter.
(iii) The reduction and oxidation potentials of dyes and *n*-butyltriphenylborate salt were measured by cyclic voltammetry. An Electroanalytical MTM System model EA9C-4z (Cracov, Poland), equipped with small-volume cell, was used for the measurements. A 1-mm platinum disk electrode was used as the working electrode. A Pt wire constituted the counter electrode, and an Ag-AgCl electrode served as the reference electrode. The supporting electrolyte was 0.1 M tetrabutylammonium perchlorate in dry acetonitrile. The solution was degassed by bubbling argon gas through the solution. The potential was swept from −2.0 to 2.0 V with the sweep rate of 500 mV/s to record the current-voltage curve.
(iv) Photoinitiated polymerization rate (*R*_p_) profiles were determined by a differential scanning calorimetry (DSC), under isothermal conditions at room temperature using a photo-DSC apparatus constructed on the basis of a TA Instruments DSC 2010 Differential Scanning Calorimeter. The 40 mg of sample was polymerized in open aluminum pans having a diameter of 6.6 mm. The irradiation of the polymerization mixture was carried out using the visible emission (488 nm) of an argon-ion laser (Melles Griot). The average power of irradiation was 15 mW/0.196 cm^2^ at 488 nm.

A polymerization solution was composed of 1 ml of MP and 9 ml of TMPTA. The co-initiator and sensitizer concentration was 5 × 10^−3^ M. As a reference sample, a polymerizing mixture containing cyanine halide (dye without a co-initiator) was used. The polymerizing mixture was not deaerated.

### Synthesis

**Scheme 1:**
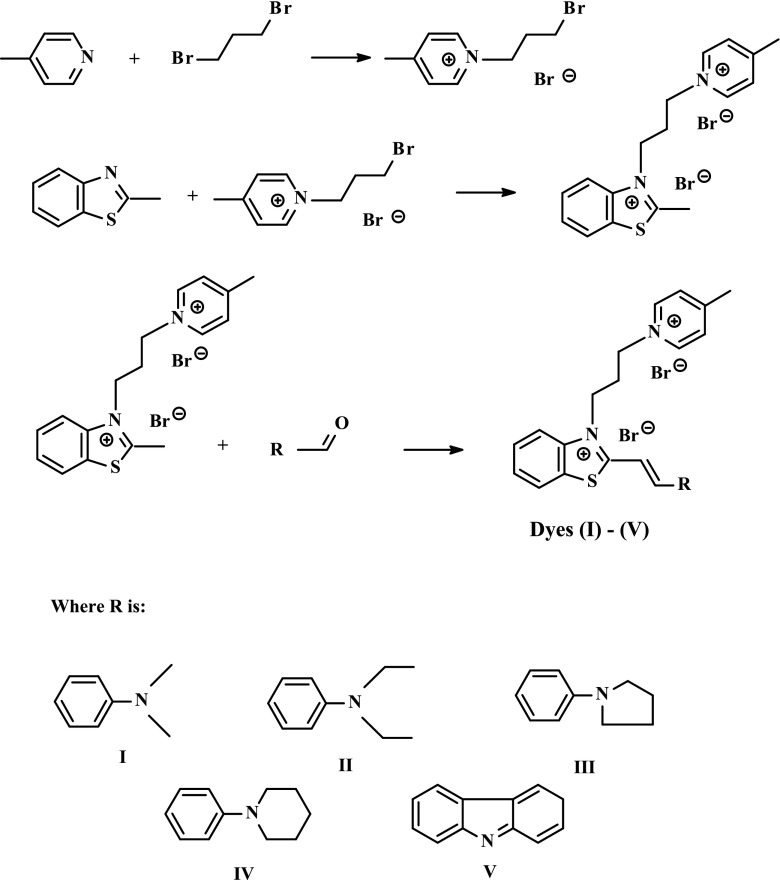
The synthesis of *N*-[3-(4-methylpyridino)propyl]-2-(*p*-substituted styryl)benzothiazolium dihalides

The synthetic approaches applied are outlined below and are based on the condensation reaction of 1-[3-(2-methylbenzothiazolium)-3-(4-methylpyridinium)]propane dibromide with various aldehydes. A general route for the synthesis of bicationic monochromophoric hemicyanine dyes is shown in Scheme 1.

As it is shown in Scheme 1, the synthesis of bicationic monochromophoric hemicyanine dyes based on 2-methylbenzothiazole undergoes via three steps:

1. The quaternization reaction of 4-methylpyridine with 1,3-dibromopropane giving *N*-(3-bromopropyl)-4-methylpyridinium bromide
2. The quaternization reaction of 2-methylbenzothiazole with *N*-(3-bromopropyl)-4-methylpyridinium bromide leading to the formation of 1-[3-(2-methylbenzothiazolium)-3-(4-methylpyridinium)]propane dibromide
3. The condensation reaction of 1-[3-(2-methylbenzothiazolium)-3-(4-methylpyridinium)]propane dibromide with appropriate aldehydes (4-(*N*,*N*-dimethylamino)benzaldehyde, 4-(*N*,*N*-diethylamino)benzaldehyde, 4-(1-pyrrolidinyl)benzaldehyde, 4-(1-piperidinyl)benzaldehyde, formylcarbazole) yielding the corresponding bicationic monochromophoric hemicyanine dye (*I*)–(*V*)

### General procedure for the synthesis of bicationic monochromophoric hemicyanine dyes (*I*)–(*V*)

*N*-(3-Bromopropyl)-4-methylpyridinium bromide was prepared based on the method described by Colichman [10]. 1-[3-(2-Methylbenzothiazolium)-3-(4-methylpyridinium)]propane dibromide was prepared using a method described by Gadjev et al. [11]. *N*-[3-(4-Methylpyridinol]-2-(*p*-*N*,*N*-dimethylaminostyryl)benzothiazolium diiodide (*I*), *N*-[3-(4-methylpyridino)propyl]-2-(*p*-*N*,*N*-diethylaminostyryl)benzothiazolium dibromide (*II*), *N*-[3-(4-methylpyridino)propyl]-2-(*p*-pyrrolidinylstyryl)benzothiazolium diiodide (*III*), *N*-[3-(4-methylpyridino)propyl]-2-(*p*-piperidinylstyryl)benzothiazolium diiodide (*IV*), and 1-*N*-[3-(4-methylpyridino)propyl-2-benzothiazolo]-2-carbazoloethane dibromide (*V*) were obtained by refluxing of 1 mmol of 1-[3-(2-methylbenzothiazolium)-3-(4-methylpyridinium)]propane dibromide with 1 mmol of appropriate aldehyde in methanol (25 ml) in the presence of few drops of piperidine for 2–8 h. If the precipitate did not form after cooling, a triplet excess of saturated aqueous/methanol solution of potassium iodide was added to the hot reaction mixture. The dye obtained was filtered and crystallized from ethanol or methanol.

### Synthesis of *N*-[3-(4-methylpyridino)propyl]-2-(*p*-*N*,*N*-dimethylaminostyryl)benzothiazolium diiodide (*I*)

The dye was prepared by the procedure described above using the mixture of 1-[3-(2-methylbenzothiazolium)-3-(4-methylpyridinium)]propane dibromide (0.0134 mol, 5.94 g) and 4-(*N*,*N*-dimethylamino)benzaldehyde (0.0134 mol, 1 g) in 25 ml of methanol with five drops of piperidine. A triple excess of saturated solution of potassium iodide in methanol was added to the hot reaction mixture. The green solid obtained was crystallized from methanol: 6.35 g, yield 70.36 %, and m.p. 249–250 °C.

^1^H NMR (DMSO-*d*_*6*_) δ (ppm): 2.037–2.076 (m, 2H, –CH_2_–); 2.585 (s, 6H, –CH_3_); 3.123 (s, 6H, –N(CH_3_)_2_); 4.790–4.838 (2H, N^+^–CH_2_–); 4.867–4.966 (d, 2H (benzothiazole)); 6.759–6.865 (d, 2H, Ar); 7.575 (d, 1H, –CH=); 7.651–7.815 (m, 2H, Ar); 7.985–8.002 (m, 3H, Ar); 8.224–8.297 (d, 1H, –CH=); 8.954–8.987 (d, 2H, (Pyr)).

Elemental analysis: Anal. Calcd. for C_26_H_29_N_3_SI_2_: C, 46.64 %; H, 4.33 %; N, 6.28 %. Found: C, 46.71 %; H, 4.02 %; N, 6.21 %.

### Synthesis of *N*-[3-(4-methylpyridino)propyl]-2-(*p*-*N*,*N*-diethylaminostyryl)benzothiazolium dibromide (*II*)

The dye was synthesized by applying the methodology described for dye (*I*) using 1-[3-(2-methylbenzothiazolium)-3-(4-methylpyridinium)]propane dibromide (0.0134 mol, 5.94 g) and 4-(*N*,*N*-diethylamino)benzaldehyde (0.0134 mol, 1.37 g) in 20 ml of methanol with five drops of piperidine as substrates. The violet solid obtained was crystallized from methanol: 4.61 g, yield 57.00 %, and m.p. 199–200 °C.

^1^H NMR (DMSO-*d*_*6*_) δ (ppm): 1.074–1.186 (t, 6H, CH_3_–); 2.484–2.581 (m, 6H, –CH_2_–); 3.493–3.528 (m, 2H, N^+^–CH_2_– (Pyr)); 4.916–4.997 (m, 2H, N^+^–CH_2_– (benzothiazole)); 6.779–6.823 (d, 1H, –CH=); 7.616–7.788 (d, 4H, Ar); 7.969–8.119 (d, 4H, Ar); 8.251–8.337 (m, 4H, Ar); 9.068–9.101 (d, 1H, –CH=).

Elemental analysis: Anal. Calcd. for C_28_H_33_N_3_SBr_2_: C, 55.72 %; H, 5.47 %; N, 6.96 %. Found: C, 55.71 %; H, 5.42 %; N, 6.91 %.

### Synthesis of *N*-[3-(4-methylpyridino)propyl]-2-(*p*-pyrrolidinylstyryl)benzothiazolium diiodide (*III*)

This dye was prepared by applying the procedure described above using 1-[3-(2-methylbenzothiazolium)-3-(4-methylpyridinium)]propane dibromide (0.0140 mol, 6.23 g) and 4-(1-pyrrolidinyl)benzaldehyde (0.0140 mol, 2.46 g) in 20 ml of methanol with five drops of piperidine. A triple excess of saturated solution of potassium iodide in methanol was added to the hot reaction mixture. The violet solid obtained was crystallized from ethanol: 6.48 g, yield 80.28 %, and m.p. 160–162 °C.

^1^H NMR (DMSO-*d*_*6*_) δ (ppm): 1.889–1.997 (t, 4H, –CH_2_–); 2.475–2.503 (m, 2H, –CH_2_–); 3.437 (s, 3H, –CH_3_); 6.548–6.671 (t, 2H, Ar); 7.290 (d, 1H, –CH=CH–), 7.464–7.529 (4H, Ar); 7.638–7.695 (d, 2H, Ar); 7.942–7.986 (d, 1H, –CH=); 7.999–8.035 (t, 2H, Pyr).

Elemental analysis: Anal. Calcd. for C_28_H_31_N_3_SI_2_: C, 48.34 %; H, 4.46 %; N, 6.04 %. Found: C, 47.99 %; H, 4.13 %; N, 6.35 %.

### Synthesis of *N*-[3-(4-methylpyridino)propyl]-2-(*p*-piperidinylstyryl)benzothiazolium diiodide (*IV*)

The dye was prepared based on the procedure described above using 1-[3-(2-methylbenzothiazolium)-3-(4-methylpyridinium)]propane dibromide (0.0067 mol, 2.93 g) and 4-(1-piperidinyl)benzaldehyde (0.0067 mol, 1.27 g) in 25 ml of methanol with five drops of piperidine. A triple excess of saturated solution of potassium iodide in methanol was added to the hot reaction mixture. The violet solid obtained was crystallized from ethanol: 3.23 g, yield 67.86 %, and m.p. 141–142 °C.

^1^H NMR (DMSO-*d*_*6*_) δ (ppm): 2.012–2.142 (m, 2H, –CH_2_–); 2.788 (s, 3H, –CH_3_); 3.442–3.521 (d, 10H, –CH_2_–); 4.537–4.993 (m, 4H, N^+^–CH_2_– (benzothiazole, Pyr)); 6.980–7.068 (d, 1H, –CH=); 7.614–7.689 (d, 1H, –CH=); 7.966–7.999 (d, 4H, Ar); 8.222–8.363 (m, 4H, Ar); 8.867–9.008 (m, 4H, Pyr).

Elemental analysis: Anal. Calcd. for C_29_H_33_N_3_SI_2_: C, 49.08 %; H, 4.65 %; N, 5.92 %. Found: C, 48.98 %; H, 4.63 %; N, 6.05 %.

### The synthesis of 1-*N*-[3-(4-methylpyridino)propyl-2-benzothiazolo]-2-carbazoloethane dibromide (*V*)

The dye was prepared according to the procedure described above using 1-[3-(2-methylbenzothiazolium)-3-(4-methylpyridinium)]propane dibromide (0.0067 mol, 2.93 g) and formylcarbazole (0.0067 mol, 1.3 g) in 25 ml of acetic acid anhydride with five drops of piperidine. The dark solid obtained was crystallized from methanol: 3.05 g, yield 73.15 %, and m.p. 182–184 °C.

^1^H NMR (DMSO-*d*_*6*_) δ (ppm): 2.479–2.598 (d, 2H, –CH_2_–); 2.874 (s, 3H, –CH_3_); 4.524–4.643 (m, 2H, N^+^–CH_2_–, (Pyr)); 5.200–5.440 (m, 2H, N^+^–CH_2_– (benzothiazole)); 7.369–7.550 (m, 2H, –CH=CH–, 8H, Ar); 7.991–8.059 (m, 4H, Ar); 8.850–8.928 (m, 4H, Pyr).

Elemental analysis: Anal. Calcd. for C_30_H_27_N_3_SBr_2_: C, 57.97 %; H, 4.35 %; N, 6.76 %. Found: C, 58.19 %; H, 4.63 %; N, 7.05 %.

## Results and discussion

### Synthesis

**Fig. 1:**
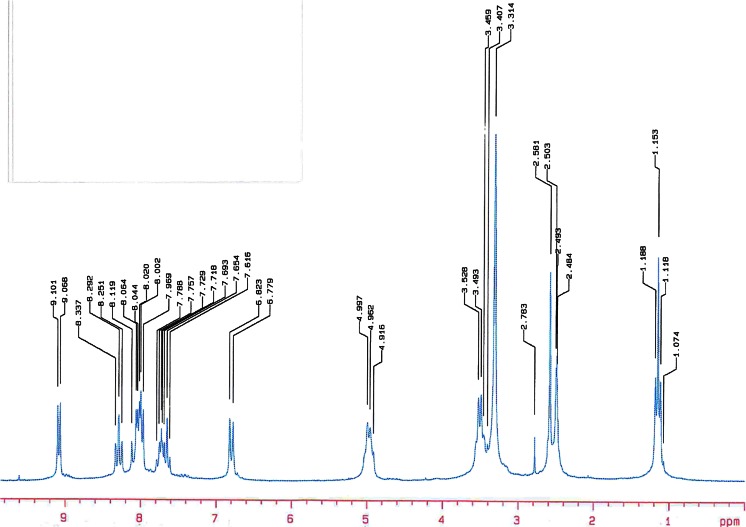
^1^H NMR spectra of *N*-[3-(4-methylpyridino)propyl]-2-(*p*-*N*,*N*-diethylaminostyryl)-benzothiazolium dibromide (*II*) in DMSO

The structures of novel bicationic monochromophoric benzothiazolestyrylium dyes are presented in Scheme 1. Details of the synthesis are given in the “Experimentalˮ section. An inspection of the experimental data shows that the melting points for all sensitizers are sharp, indicating high purity and crystalline phase of the resulting dyes. Figure 1 shows the ^1^H NMR spectra of selected hemicyanine dye (*II*) in the region of *δ* = 1.0–9 ppm. In the region, the signals characteristic for the protons in benzene ring, pyridinyl ring, and the central double bond usually are present. All the peaks are assigned to the corresponding hydrogen atoms in molecule, as presented in “Experimentalˮ section. It is noteworthy that the ^1^H NMR spectra display two characteristic doublets localized at chemical shifts in the range of 6.8–8.3 ppm. They are attributed to both vinyl hydrogen atoms.

The bands that appeared in the range from about 6.5 to 9.1 ppm are the signal for the aromatic protons. According to thin-layer chromatography analysis (silica gel 60, F-254), with the use of methanol-acetone mixture (2:1) as the eluent, the hemicyanine dyes are of high purity. Taking into account the ^1^H NMR spectra, the elemental analysis, and thin-layer chromatography results, one can conclude that purity of the dyes was as it is required for spectroscopic studies (>99 %).

### Spectroscopic properties

**Fig. 2:**
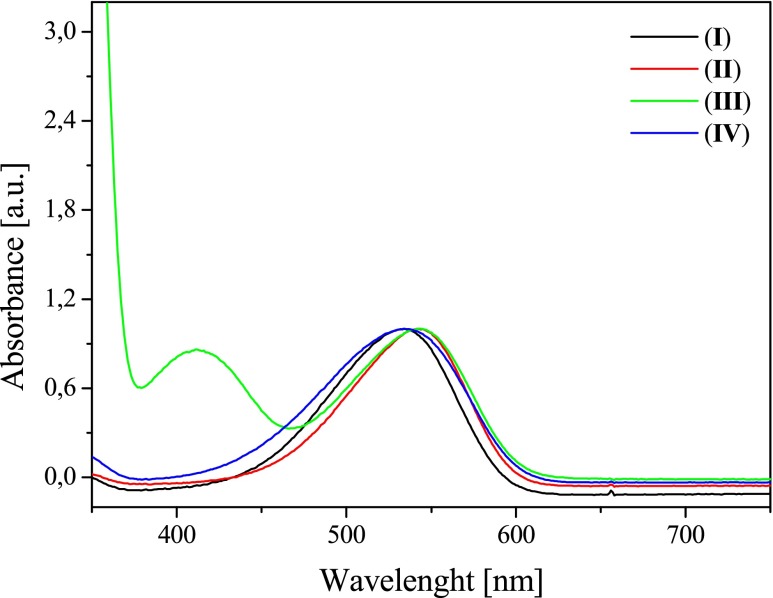
Effect of a dye structure on the electronic absorption spectra in acetonitrile solution, recorded at room temperature

**Fig. 3:**
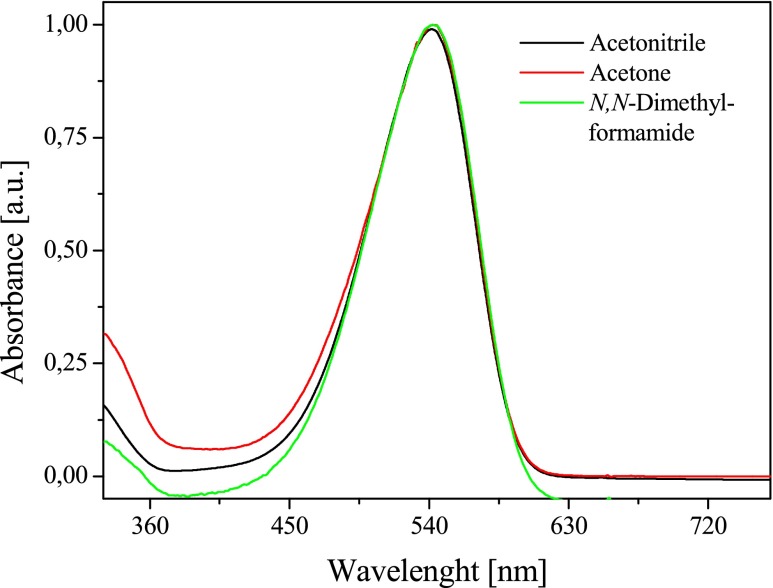
Electronic absorption spectra of *N*-[3-(4-methylpyridino)propyl]-2-(*p*-*N*,*N*-diethylaminostyryl)benzothiazolium dibromide (*II*) in different polarity solvents recorded at 293 K

The tested compounds belong to the group of asymmetric dyes, so-called hemicyanine dyes. These dyes have a D-π-A^+^X^−^ structure and differ from each other the type of substituent in the vinyl group. An asymmetric structure of compounds has an influence on the nature of both the electron absorption spectra and fluorescence spectra. The examples of electron absorption spectra of the bicationic monochromophoric hemicyanine dyes studied are shown in Figs. 2 and 3. Table 1 collects the values of the absorption maximum positions, the fluorescence maximum positions, energy of 0 → 0 transition, Stokes shifts, reduction potentials, and oxidation potentials.

**Table 1:** The spectroscopic and electrochemical properties

Dye	Solvent	*λ*_ab_ (nm)	*λ*_fl_ (nm)	*E*_00_ (ev)	Stokes shift (/cm)	*E*_red_ (eV)	*E*_ox_ (eV)
I	Acetone	531	658	2.18	3,600	−1.132	0.702
	Acetonitrile	534	605	2.14	2,200		
	DMF	534	622	2.15	2,700		
II	Acetone	545	672	2.01	3,500	−1.052	1.266
	Acetonitrile	541	615	2.15	2,200		
	DMF	542	657	2.15	3,200		
III	Acetone	542	628	2.17	2,500	−0.748	0.400
	Acetonitrile	543	618	2.15	2,200		
	DMF	544	620	2.14	2,300		
IV	Acetone	532	652	2.16	3,500	−0.712	0.384
	Acetonitrile	537	614	2.12	2,300		
	DMF	534	635	2.14	3,000		
V	Acetone	554	582	2.21	900	−1.234	0.845
	Acetonitrile	555	573	2.31	600		
	DMF	559	589	2.18	900		

The bicationic monochromophoric hemicyanine dyes show two characteristic absorption bands. The absorption band localized in the longer wavelengths spectrum region is the charge-transfer band, attributed to the intermolecular charge transfer from free electron pair of nitrogen atom on the quaternary nitrogen atom in benzothiazole moiety. The absorption band in the range from 360 to 400 nm is attributed to the π → π^*^ transition. The position of absorption bands depends on the dye structure. The dyes studied differ in the type of *N*-alkylamino group in styryl moiety. Dye I possessing *N*,*N*-dimethylamino group in styryl moiety has the maximum absorption band at 534 nm. The introduction of *N*,*N*-diethylamino group in dye structure causes in the bathochromic shift of the absorption band about 10 nm. The presence of *N*-formylcarbazole moiety (dye *V*) provides the highest absorption band shift toward the longer wavelengths (about 20 nm). The molar absorption coefficient of dyes under study ranges from 20,000 to 60,000 dm^3^/mol/cm and depends on the dye structure and solvent polarity. Based on the results presented in Table 1, it is seen that the solvent polarity only weakly affects the position of the maximum absorption band. The increase in solvent polarity causes a slight bathochromic shift of long-wave absorption band (Fig. 2, Table 1). The polarity of the solvent has an insignificant impact on the solvatochromic properties of the new dyes.

**Fig. 4:**
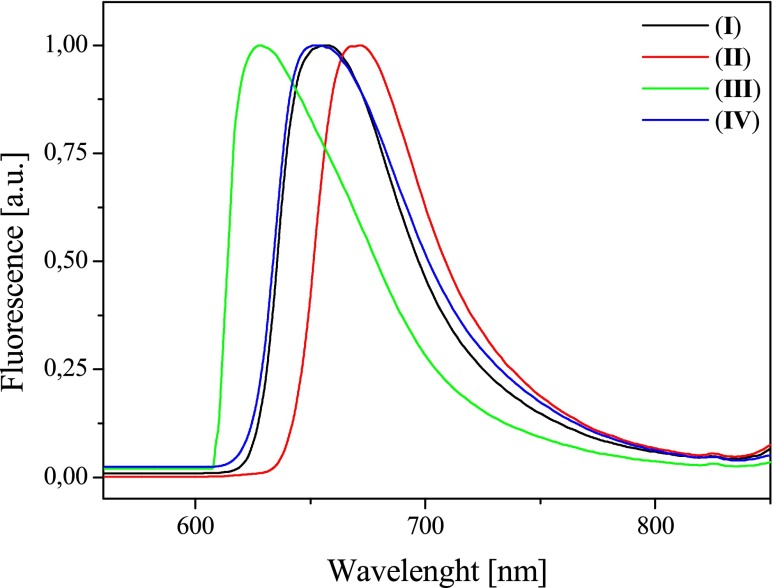
Effect of a dye structure on the fluorescence spectra in acetonitrile solution, recorded at room temperature

**Fig. 5:**
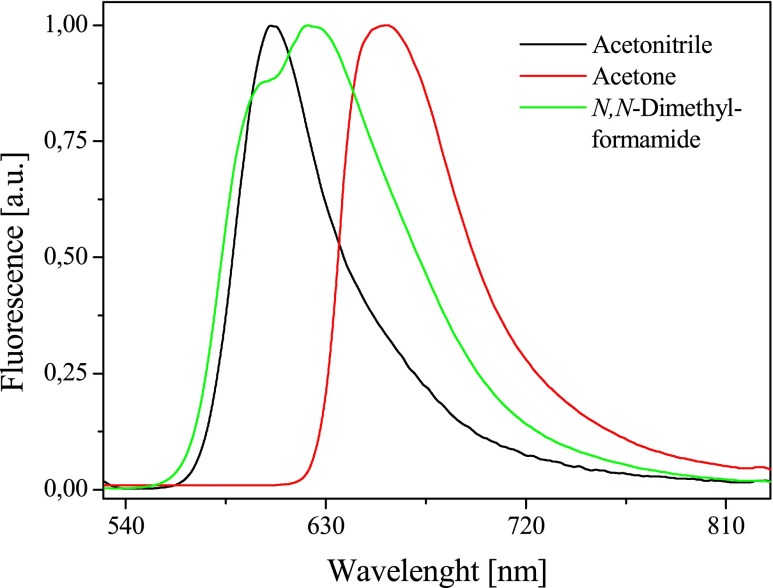
Fluorescence spectra of *N*-[3-(4-methylpyridino)propyl]-2-(*p*-*N*,*N*-dimethylaminostyryl)benzothiazolium dibromide (*I*) in different polarity solvents recorded at 293 K

The benzothiazolestyrylium dyes posses an intense fluorescence in the range from 550 to 750 nm (Figs. 4 and 5).

**Scheme 2:**
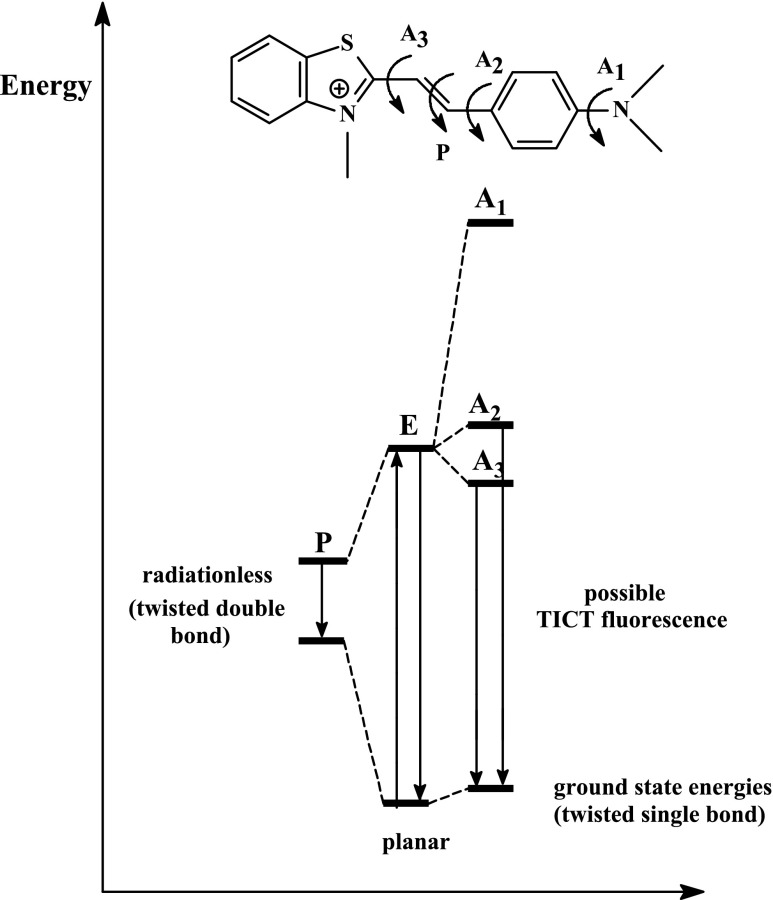
The energy levels of three different emission states and the corresponding conformations of the benzothiazolestyrylium dyes

From the study of Strehmel et al. [12] on the spectroscopic properties of monocationic styryl pyridinium dyes, it is known that dyes possessing D⋅⋅⋅π⋅⋅⋅A structure may be characterized by the presence of three fluorescence states corresponding to different geometric conformations of dye in excited state. Scheme 2 presents the energy levels of three different emission states and the corresponding conformations of the benzothiazolestyrylium dyes.

The fluorescence spectra of dyes tested show the presence of long-wave band with a maximum in the range from 580 to 670 nm and the second emission band in the blue region of the spectrum at about 580 nm. Based on the energy levels shown in Scheme 2, it may be concluded that the long-wave band is an evidence of the presence of the lowest energy conformers, e.g., E or A_3_ (planar conformation or twisted single bond). By contrast, the presence of a second emission band is a result of the rotation around the C-N single bond in styryl moiety. The position of maximum of emission depends on the dye structure. Dye *I* possessing the *N*,*N*-dimethylamino group in styryl moiety has the maximum of fluorescence at 605, 658, and 622 nm. The introduction of *N*,*N*-diethylamino group in dye structure causes in the bathochromic shift of the fluorescence band about 10–30 nm. The type of solvent affects on the position of the fluorescence band.

### Determination of the energy of transition 0 → 0

**Fig. 6:**
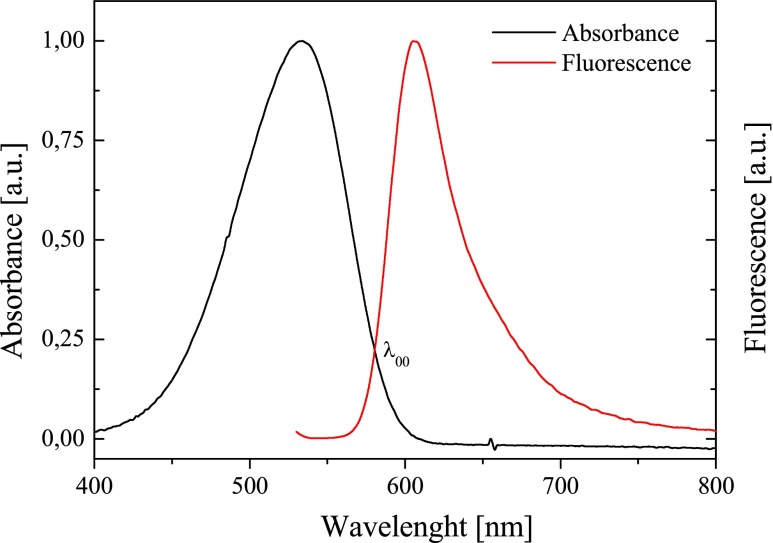
The normalized absorption and fluorescence spectra of *N*-[3-(4-methylpyridino)propyl]-2-(*p*-*N*,*N*-dimethylaminostyryl)benzothiazolium dibromide (*I*) in acetonitrile

In order to determine the energy transition (singlet-state energy *E*_00_) between the lowest ground state and the lowest excited state, it was necessary to overlap both normalized absorption and fluorescence spectra of new sensitizers. Figure 6 presents the normalized absorption and fluorescence spectra of selected dye in acetonitrile as a solvent. The fluorescence spectra of the dyes tested are not a mirror image of the absorption spectra. Based on this, one can conclude that the geometry of sensitizer in both ground and excited states is different.

The values of energy *E*_0 → 0_ are presented in Table 1. The difference in energy between the absorbed and emitted radiation is known as the Stokes shift [13]. The Stokes shift values obtained for novel bicationic monochromophoric benzothiazolestyrylium dyes are shown in Table 1. The Stokes shift of most of dyes tested is in the range from 2,000 to 3,600/cm. The highest value of Stokes shift (>3,000/cm) is observed in acetone as a solvent. The high values of Stokes shift suggest the difference in the dye geometrical structure in ground and excited states.

### Electrochemical properties

**Fig. 7:**
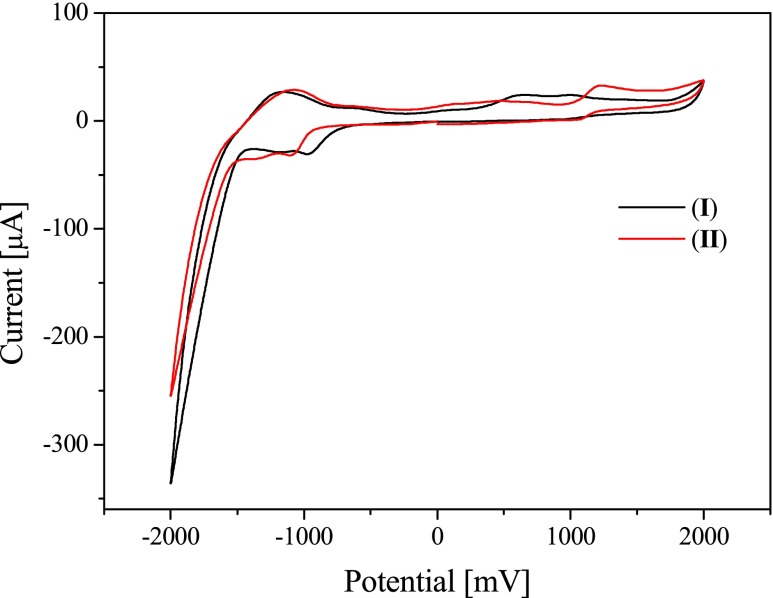
Cyclic voltammograms recorded for *N*-[3-(4-methylpyridino)propyl]-2-(*p*-*N*,*N*-dimethylaminostyryl)benzothiazolium diiodide (*I*) and *N*-[3-(4-methylpyridino)propyl]-2-(*p*-*N*,*N*-diethylamino-styryl)benzothiazolium dibromide (*II*) in 0.1 M tetrabutylammonium perchlorate solution in dry acetonitrile as the supporting electrolyte

The electrochemical reduction of the dyes is reversible, as it is shown in Fig. 7.

In such dye-based photoinitiator systems, the initiating radicals are generated via photoinduced electron-transfer process [14]. Therefore, intermolecular electron transfer between the excited dye and organoborate salt must be thermodynamically allowed. Taking this into account, the values of free energy changes for electron-transfer process (Δ*G*_el_), calculated from the Rehm-Weller equation (Eq. 1), must be negative [15].

1 $$ \varDelta {G}_{e l}={E}_{ox}\left( E/{E}^{\bullet}\right)-{E}_{r ed}\left( Dye/ Dy{e}^{\bullet}\right)-{E}_{00}(Dye)-\frac{Z_1{Z}_2}{\varepsilon {r}_{12}} $$

The value of Δ*G*_el_ was calculated using the oxidation potential of *n*-butyltriphenylborate salt (*E*_ox_ = 1.16 eV), the singlet excited state energy (*E*_00_(S)) of the dye (Table 1) and the reduction potential *E*_red_(Dye/Dye^•^) of the sensitizers (see data in Table 1). The Coulombic energy coefficient (*Z*_1_ *Z*_2_/εr_12_) was omitted in these calculations because the neutral radicals of borate salt, as well as sensitizers, were formed in the electron-transfer process. The *E*_ox_ and *E*_red_ of both photoredox pair components were determined from cyclic voltammetry measurements (Table 1).

The oxidation of tetramethylammonium *n*-butyltriphenylborate is dissociative, and this in turn causes the electrochemical process irreversible. The thermodynamically meaningful oxidation potential can be established using an indirect, kinetic method described by Murphy and Schuster [15]. Oxidation potentials measured electrochemically and kinetically differ by about 0.3 V. Therefore, the obtained electrochemical values for both components of photoredox pairs may have only approximate thermodynamic meaning, yet allow us to estimate roughly the free energy of activation (Δ*G*_el_) for the photoinduced electron-transfer process.

The measured values of the reduction potential of sensitizers, the oxidation potential of the electron donor (*E*_ox_ = 1.16 eV), and the singlet-state energy of the dyes allow us to calculate the free energy change for the photoinduced intermolecular electron-transfer process. The estimated data are summarized in Table 2.

**Table 2:** Thermodynamic parameters for dyes (*I*)–(*V*)

Dye	Δ*G*_el_ (eV)	Heat flow (mW)	*R*_p_ (/μmols)	Double bond conversion (%)	
				After 1 min of irradiation	After 2 min of irradiation
I	0.152	128	1.63	14.02	15.65
II	0.062	84	1.07	10.50	11.35
III	−0.242	34	0.437	4.75	7.22
IV	−0.248	57	0.727	6.70	7.91
V	0.0849	112	1.432	13.50	16.70

The reduction potential of tetramethylammonium *n*-butyltriphenylborate is equal to 1.16 V

The values of Δ*G*_el_ for tested photoinitiating systems oscillate in the range from −0.248 to 0.152 eV. Taking this into account, the thermodynamic parameters presented in Table 2, it is seen that all combinations of benzothiazolestyrylium dyes/borate salt photoinitiating systems were found to possess small driving forces of electron-transfer process (Δ*G*_el_ > −0.248 eV) upon exposure to light. The estimated, according the Rehm-Weller equation, values of free energy activation for the electron-transfer process from borate anion to the excited hemicyanine dyes show that for tested photoredox pairs, the electron-transfer process is thermodynamically allowed (negative values of Δ*G*_el_) only in the case of the following photoinitiators: *IIIB* and *IVB*. Basing on this, only these photoinitiators should initiate free-radical polymerization. But, the kinetic results obtained and presented in Table 2 do not confirm the thermodynamical requirement. Therefore, one should conclude that there are other factors which may effect on the overall rate of free-radical polymerization.

If the electron transfer is not diffusion controlled, it could be the rate-determining step for the polymerization. In such a case, the polymerization rate would increase with the increase in −Δ*G*_el_ values, at least up to the diffusion-controlled limit. The rate of electron transfer cannot be the rate-determining step if it approaches the diffusion-controlled limit (then it becomes independent of Δ*G*_el_). In such a situation, other factors (for example, the reactivity of the primary radicals) would control the polymerization rate. Thus, the efficiency of any PET mechanism decreases as the viscosity of the polymerizing medium increases, and the formation of reactive radicals by the PET mechanism may be effective only at the initial time of polymerization.

### Photoinitiating abilities

Five photoredox pairs, consisting of benzothiazolestyrylium dyes (*I*)–(*V*) (acting as electron acceptors) and *n*-butyltriphenylborate salt (acting as electron donor), were tested as photoinitiating systems for free-radical polymerization of TMPTA.

To transform the obtained hemicyanine dyes into efficient free-radical polymerization initiating systems, an anion exchange from halide to borate was necessary. The ion exchange reaction was performed using the procedure given by Damico [9]. The final products were identified by ^1^H NMR. The spectra and results obtained were the evidence that the tested salts were of desired structures.

**Fig. 8:**
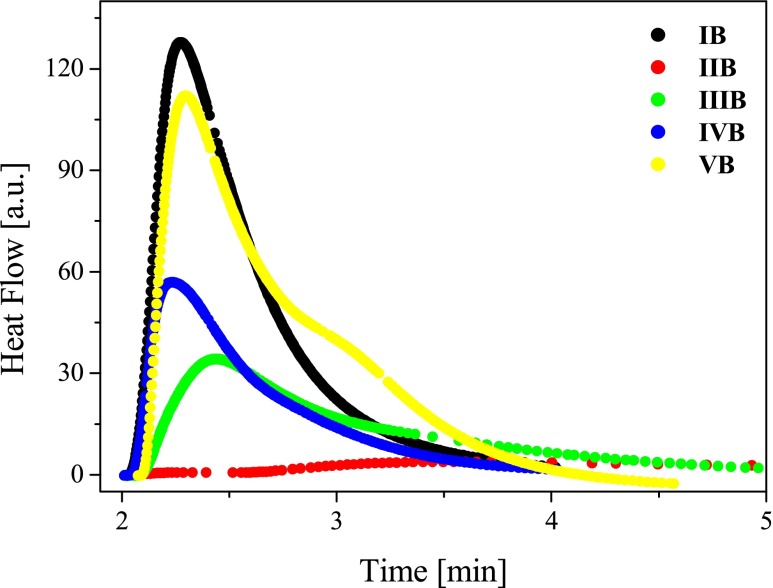
The family of kinetic curves recorded during the measurements of the flow of heat for the photoinitiated polymerization of the TMPTA/MP (9/1) mixture initiated by benzothiazolestyrylium borate salts. The concentration of photoinitiating systems was equal 5 × 10^−3^ M, *I*_a_ = 76 mW/cm^2^

Since the maximum of absorption band of tested dyes is located at about 530 nm for the photoinitiation of polymerization, the emission of an argon-ion laser emitting at 488 and 514 nm (Meles Griot) was used. Preliminary experiments show that TMPTA does not polymerize at 488 nm if any one component of the photoinitiating system was missing. The kinetic curves obtained during the polymerization of TMPTA/MP (9/1) mixture employing benzothiazolestyrylium dyes borate salts, under irradiation with a visible light, are shown in Fig. 8.

The rates of photoinitiated polymerization measured for all tested photoredox pairs are collected in Table 2. The initiation of polymerization via photoinduced intermolecular electron-transfer process involves many steps. Those include PET from an electron donor to an excited state of a dye or from an excited electron donor to the ground state of an electron acceptor followed by secondary reactions yielding a neutral radical initiating polymerization.

The step determining the reaction rate of the free-radical-initiated polymerization via intermolecular electron-transfer process (PET) is dependent on the nature of the dye and the electron donor (or acceptor). In the case of alkyltriphenylborate anion acting as an electron donor, the alkyl radical is formed as a result of rapid cleavage of the alkylboron bond within the boranyl radical produced after an electron transfer [5, 6]. According to the study Chatterjee’s et al studies on symmetrical cyanine borate initiators in nonpolar or medium polarity solvents, one can treat cyanine cation and borate anion as an ion pair. However, our studies on the influence of co-initiator concentration on the rate of photoinitiated polymerization show a distinct increase in the rate of polymerization as the concentration of borate anion increases for an identical monomer-dye formulation [14, 17, 18]. This finding suggests that at the concentration of borate anion equal to the concentration of dye cation, only a part of photoredox pairs exists as ion pairs. Since the electron transfer in cyanine dyes occurs in their singlet state, the existence of a cyanine cation and borate anion as ion pair is the basic prerequisite for the effective electron transfer. It is obvious that the additional amount of borate anion in the polymerizing composition should shift the equilibrium between free ion-ion pairs to a higher ion pair concentration and cause an increase in the photoinitiation efficiency of the dye-borate salt. It seems to be obvious that an artificial increase of the number of electron donor moieties within one molecule achieved by the coupling of the second borate anion to the dye molecule should improve the photoinitiating ability. Our experiments confirm this notion.

**Fig. 9:**
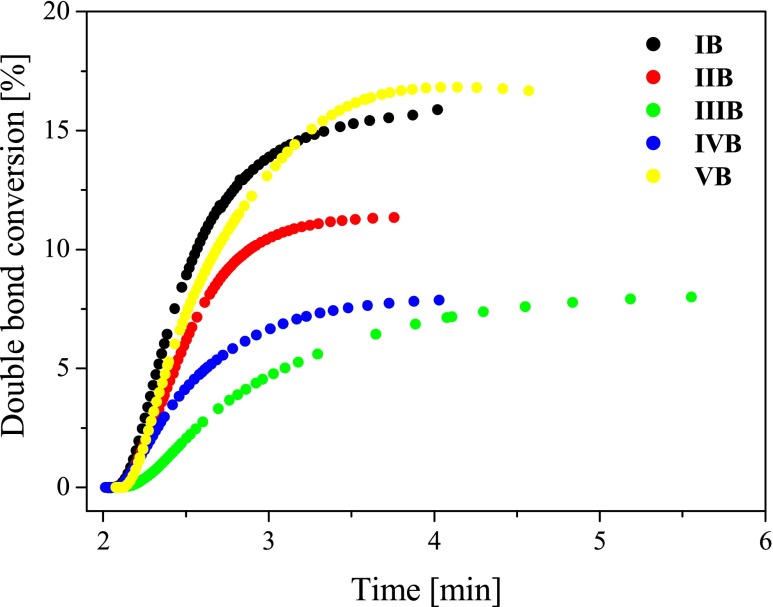
Relationship between the degree of double bond (–CH=CH–) conversion of trimetylolpropane triacrylate and irradiation time. The type of photoinitiating system is marked in the figure

The data presented in Table 2 and Fig. 8 clearly indicate that the efficiency of TMPTA polymerization depends on the sensitizer structure. The highest efficiency for TMPTA free-radical polymerization was obtained for sensitizer possessing *p*-(*N*,*N*-dimethylamino)phenyl group, whereas the lowest efficiency was observed in the case of *p*-(pirydyno)phenyl substituent. These results are related to different reduction potentials (*E*_red_) of hemicyanine dyes (see data presented in Table 1). Specifically, the dyes (*I*) and (*V*) can be readily reduced in the presence of co-initiator used, in comparison with dyes (*III*) and (*IV*) due to their lower reduction potentials. Therefore, the photoinitiating systems that contain *IB* have the highest values for photoinduced electron transfer Δ*G*_el_ (Table 2). The degree of conversion of the monomer into polymer also depends on the sensitizer structure (Fig. 9).

The values of degree of TMPTA conversion determined after a specific irradiation time are presented in Table 2. From the data obtained during the measurements, it was deducted that the conversion of monomer ranges from 4.75 to 16.7 %.

Another interesting feature of the tested new heterobicationic benzothiazolestyrylium dyes tested is worthy of attention. Analyzing the data summarized in Table 2, one can find that the type of substituent present in the styrylium moiety attached to the benzothiazole ring causes a significant variation in the photoinitiating abilities of the bicationic hemicyanine borates. The sensitizers possess asymmetrical structure that consists of an electron-donating moiety and a heterocyclic moiety, which can act as an electron-deficient acceptor. Furthermore, donor and acceptor are in direct electronic conjugation via a vinyl π-system, and the acceptor moiety is charged [13]. We tested asymmetrical cyanine dyes with electron donor-acceptor moieties on the opposite sides of the vinyl group with additionally bonded pyridinium ring. The electron-donating moiety possesses various substituents in *para* position of phenyl ring (except dye (*V*)). On the basis of our experiments, it appears that the initiators possessing the dimethylamino, *N*-formylcarbazole and diethylamino groups in *para* position of phenyl ring (*IB*, *VB*, *IIB*), initiate the free-radical polymerization with the highest rates. For the cationic photoinitiating systems, the photoinitiating ability decreases in the following order:

$$ IB> VB> IIB> IVB> IIIB $$

This suggest that the presence of –*N*(CH_3_)_2_, –*N*-formylcarbazole, or /–*N*(C_2_H_5_)_2_ in styrylium moiety causes an enhancement of the electron donor concentration (borate anion) in the proximity to the absorbing dye. In summary, their photoinitiating ability is comparable or lower than the photoinitiating ability of well-known commonly used photoinitiators operating in the visible-light region [1, 8, 13, 16–18].

**Fig. 10:**
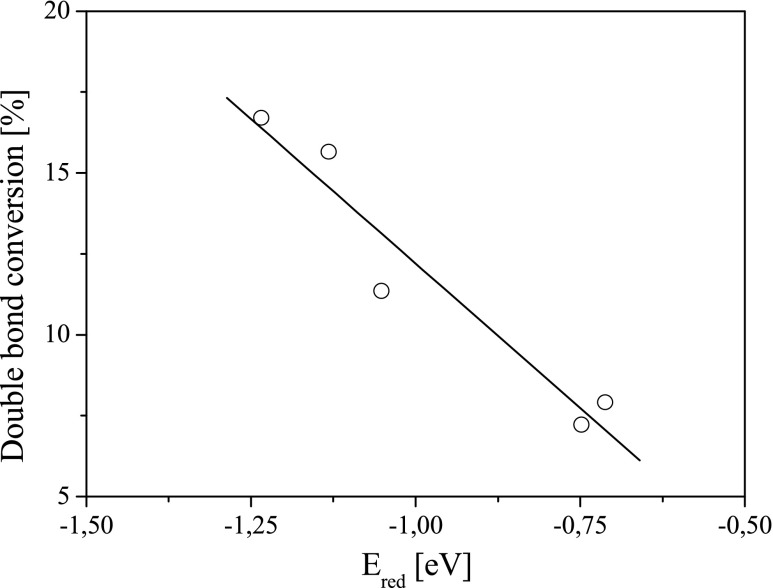
The relationship between the degree of double bond conversion and reduction potential (*E*_red_) of sensitizers

**Fig. 11:**
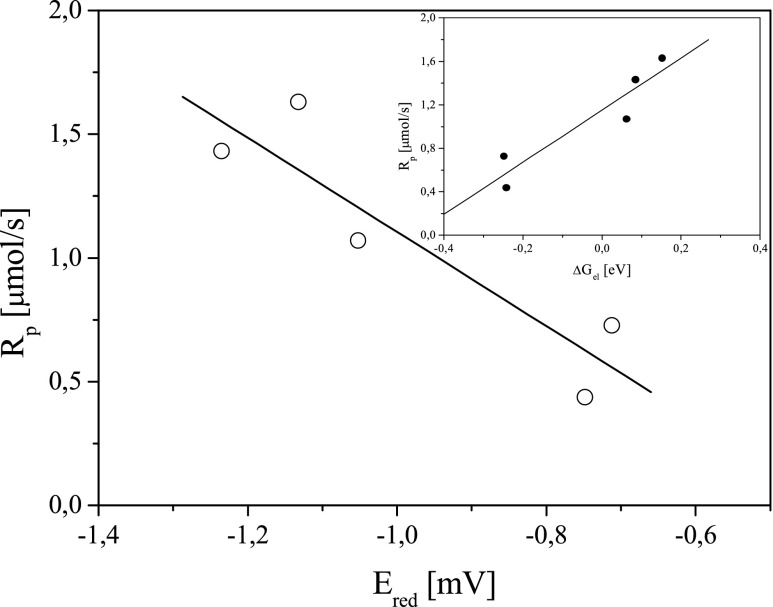
The relationship between the rate of polymerization and reduction potential (*E*_red_) of sensitizers. *Inset* is the relationship between the rate of polymerization and free energy change for electron-transfer process (Δ*G*_el_)

The degree of monomer conversion and also the rate of polymerization depend on both the structure and reduction potential of the dye (Figs. 10 and 11).

**Fig. 12:**
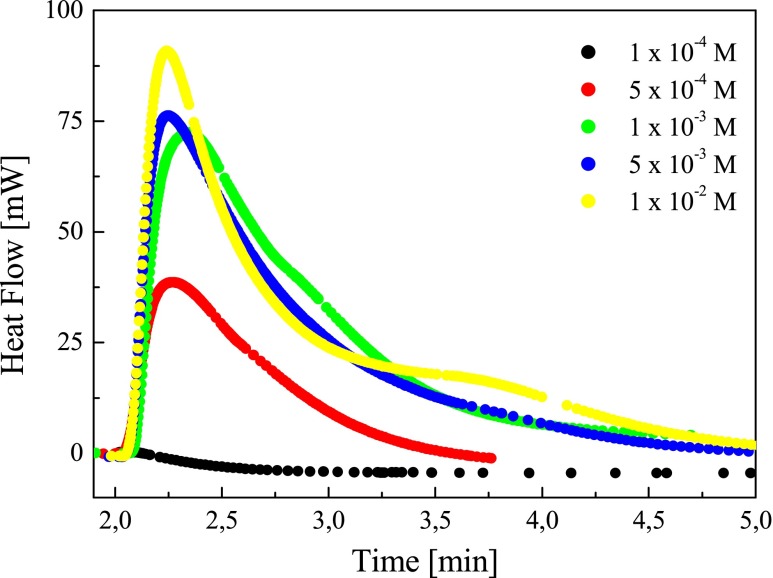
The family of the kinetic curves recorded during the measurements of the flow of heat for the photoinitiated polymerization of TMPTA/MP (9/1) mixture, initiated by *N*-[3-(4-methyl-pyridino)propyl]-2-(*p*-*N*,*N*-dimethylaminostyryl)benzothiazolium bis(*n*-butyltriphenylborate) (*IB*) at its different concentrations, *I*_a_ = 76 mW/cm^2^

The concentration of the photoinitiating system itself also plays a key role in photopolymerization. In a conventional UV-Vis photopolymerization, *R*_p_ increases with the increase of concentration of the initiator up to a certain value, than it decreases. This behavior is attributed to the “internal filter effect.” This becomes more significant for photoinitiators with high molar absorption coefficient [19]. The kinetic curves recorded for photoinitiated polymerization at different photoinitiator concentrations are presented in Fig. 12.

**Fig. 13:**
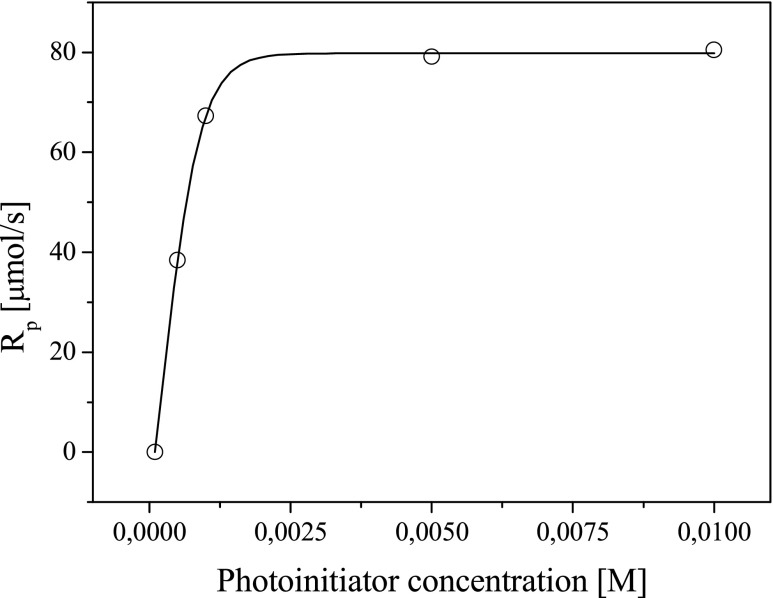
Rate of polymerization versus photoinitiator concentration

Data in Fig. 12 shows that the time needed to reach the curve maximum decreases when the initiator (*IB*) concentration increases. The observed property is related to the rate of heat evolution in the initial time of polymerization. Figure 13 presents the relationship between the rate of polymerization and the photoinitiator concentration.

It is evident that as photoinitiating system concentration increases, the rate of polymerization increases and reaches a maximum. The highest rates of polymerization (for 3-mm thick sample) were achieved at an initiator concentration of about 5 × 10^−3^ M for the tested photoinitiating systems with two electron donors in one molecule.

## Conclusions

The spectroscopic and electrochemical properties of bicationic hemicyanine dyes tested are similar to those of their monocationic analogs. Therefore, these compounds paired with organic borate salts can act as an absorber of the light in visible-light photoinitiators for acrylate monomer polymerization. The introduction to the monocationic hemicyanine dye with the additional organic cation, *p*-methylpyridinium moiety, artificially increased concentration of the *n*-butyltriphenylborate anion (acting as an electron donor) in proximity to the electron accepting excited hemicyanine dye chromophore. The artifical increase of an electron donor concentration in hemicyanine chromophore proximity causes an enhancement of the photoinitiating abilities of the bicationic photoredox pairs.
